# Simultaneous regression of non‐small cell lung cancer and orbital extranodal marginal zone lymphoma with chemoradiotherapy for lung cancer

**DOI:** 10.1002/rcr2.1171

**Published:** 2023-05-30

**Authors:** Issei Oi, Zentaro Saito, Takanori Ito, Takuma Imakita, Osamu Kanai, Kohei Fujita, Hiromasa Tachibana, Minako Mori, Koki Moriyoshi, Tadashi Mio

**Affiliations:** ^1^ Division of Respiratory Medicine Center for Respiratory Diseases, National Hospital Organization Kyoto Medical Center Kyoto Japan; ^2^ Department of Hematology National Hospital Organisation Kyoto Medical Center Kyoto Japan; ^3^ Department of Pathology National Hospital Organisation Kyoto Medical Center Kyoto Japan

**Keywords:** chemotherapy, extra marginal zone lymphoma, lung cancer, orbital lymphoma

## Abstract

This report describes the case of a 79‐year‐old Japanese man diagnosed with orbital extranodal marginal zone lymphoma (EMZL) and stage IIIA lung cancer. The patient received concurrent chemoradiation for lung cancer with carboplatin/paclitaxel treatment, resulting in regression of both the lymphoma and lung cancer. To our knowledge, this is the first reported case of concurrent orbital EMZL and lung cancer. In this case, a treatment strategy that prioritized lung cancer treatment was deemed appropriate. This case suggests that chemotherapy with carboplatin and paclitaxel may serve as an effective treatment for both lung cancer and lymphoma.

## Graphical abstract

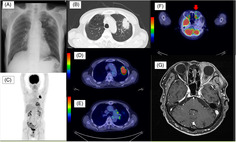

## INTRODUCTION

Concurrent cancers are frequently observed, and treatment strategies should be individualized based on the prognosis of the overlapping diseases and their rate of progression. Herein, we present the case of a patient with an orbital extranodal marginal zone lymphoma (EMZL) of mucosa‐associated lymphoid tissue, a low‐grade lymphoma, and a stage IIIA lung squamous cell carcinoma. To our knowledge, this is the first reported case of concurrent orbital EMZL and lung cancer. Concurrent chemoradiation for lung cancer resulted in the regression of both lymphoma and lung cancer.

## CASE REPORT

**FIGURE 1:**
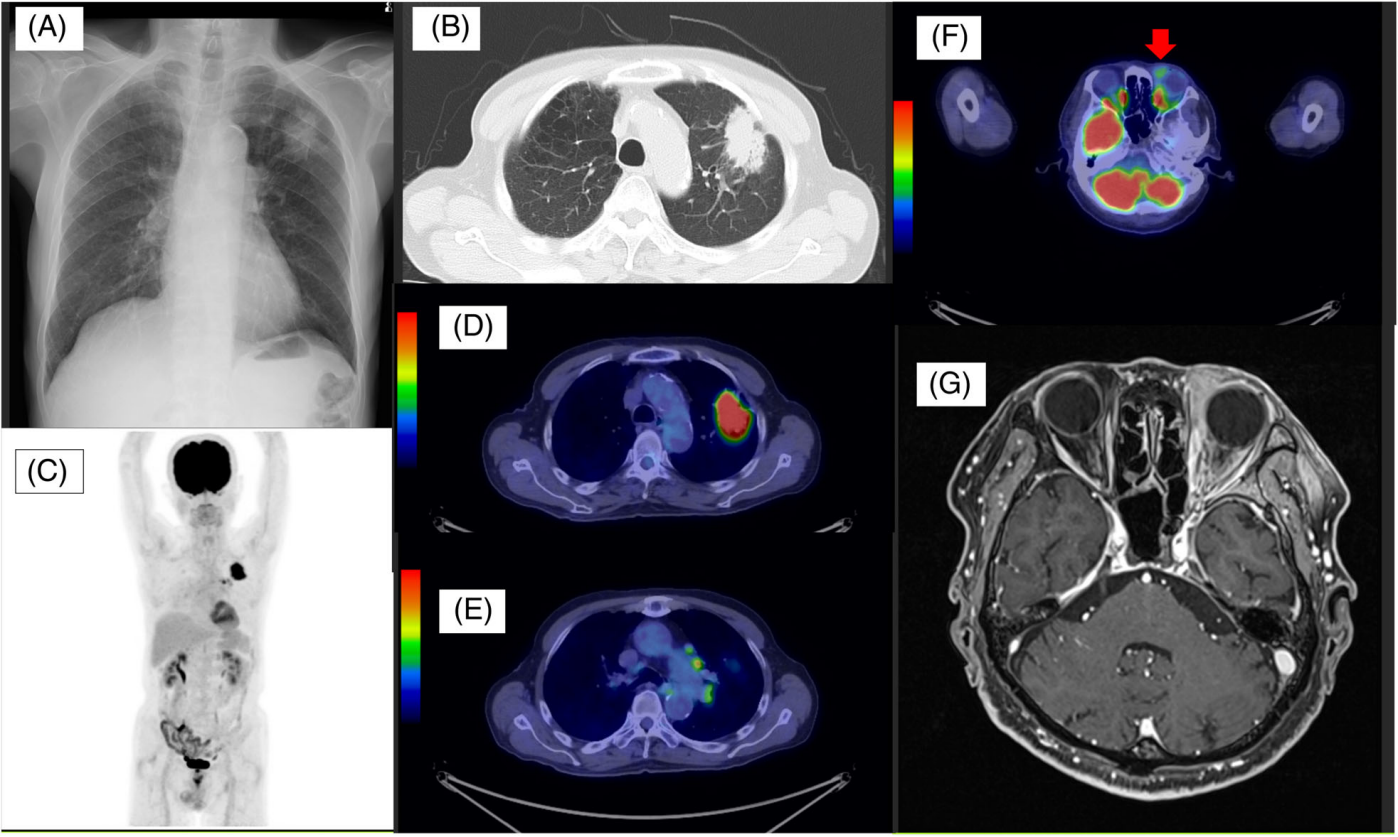
Radiographic findings on the diagnosis. (A) Chest radiograph at the initial visit to our hospital. (B) Primary lung cancer in the left upper lobe, revealed by computed tomography. (C) Overview of fluorodeoxyglucose F18 positron emission tomography (FDG PET). (D, E) FDG accumulation in the primary lung cancer and mediastinal lymph nodes on FDG PET. (F) Orbital mass on FDG PET. (G) Orbital mass on contrast‐enhanced MRI.

**FIGURE 2:**
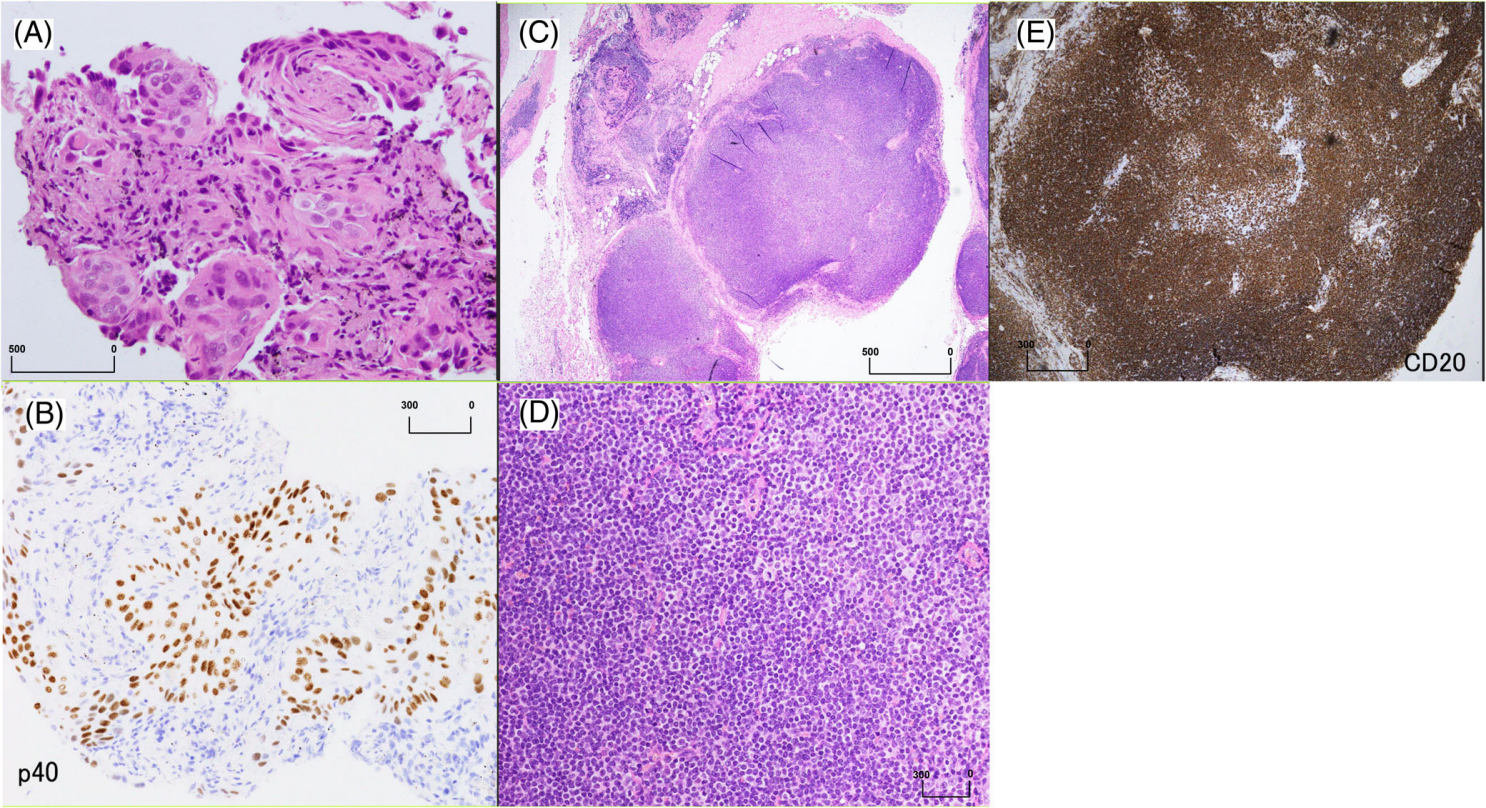
Pathological diagnosis. (A, B) Pathological examination of a transbronchial lung biopsy revealing tumour cells growing on the foci and intercellular bridges. Immunostaining is p40‐positive and TTF‐1‐negative. (C–E) Multinodular proliferation of small lymphocytes with nuclei nodules within fibrous connective tissue, forming a mass. The immunophenotype is positive for CD20 and negative for CD3, CD5, CD10, CD23, and cyclin D1. Flow cytometry showing light chain restriction with a predominance of κ chains. (A) Lung biopsy. Haematoxylin and eosin (HE) stain. (B) Lung biopsy. p40 stain. (C, D) Soft tissue biopsy of the orbit. HE staining, low‐power (C), and high‐power fields (D). (E) Soft tissue biopsy of the orbit. CD 20 stain.

**FIGURE 3:**
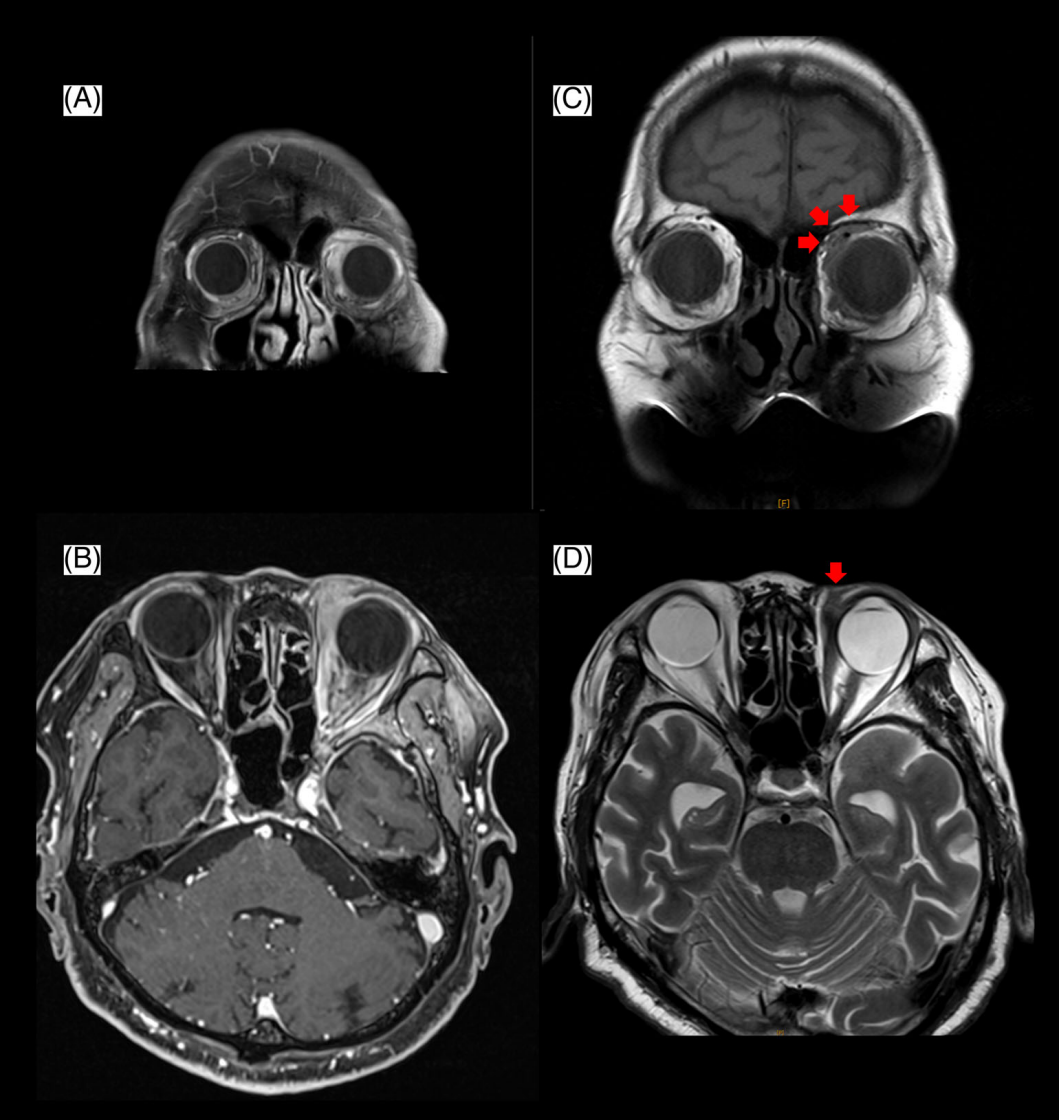
Magnetic resonance imaging (MRI) finding of orbital extranodal marginal zone lymphoma (EMZL) 1 month after concurrent chemoradiotherapy for lung cancer. (A, B) Orbital EMZL at diagnosis on enhanced MRI. (C, D) Orbital mass EMZL 1 month after chemotherapy on plain MRI.

A 79‐year‐old Japanese man presented to the Department of Respiratory Medicine at our hospital because of a chest radiographic abnormality (Figure 1A). He had no specific respiratory symptoms but complained of difficulty in opening his left eye. Computed tomography (CT) and fluorodeoxyglucose F18 positron emission tomography (FDG PET) scans revealed a 4.0 cm × 2.4 cm mass in the left upper lobe and mediastinal lymphadenopathies; no distant metastases were observed (Figure 1B–E). Additionally, an orbital mass was detected by FDG PET and contrast‐enhanced magnetic resonance imaging (MRI) of the head (Figure 1F, G). Histopathological examination of the transbronchial lung biopsy revealed squamous cell lung carcinoma at clinical stage cT3N2M0 (Figure 2A, B), and percutaneous biopsy of the orbital mass revealed EMZL (Figure 2C–E). An additional bone marrow biopsy confirmed the EMZL, which was diagnosed as stage IE. The patient was initially treated with chemotherapy, carboplatin (area under the curve, 2) and paclitaxel (40 mg/m^2^) weekly, and concurrent thoracic radiation 60 Gy/30 Fr for lung cancer. One month after chemotherapy, both the lung cancer and orbital EMZL regressed (Figure 3), and the patient was able to open his left eye. Radiotherapy was planned for the orbital EMZL.

## DISCUSSION

We report a rare case of concurrent malignancies in which treatment for lung cancer was also effective for EMZL.

In Japan, lung cancer is the leading cause of cancer‐related deaths in men, with 52,600 deaths reported in 2021.^1^ Malignant lymphomas affect 19,200 men annually;^1^ most are intranodal lymphomas, whereas extranodal lymphomas are rare. Lymphomas of the ocular adnexa account for approximately 5%–10% of extranodal lymphomas,^2^ and 50%–63% of ocular adnexal lymphomas are orbital lymphomas.^2^ EMZL is the most common type of orbital lymphoma, accounting for 57% of all cases.^3^ To our knowledge, there are no reports of the simultaneous occurrence of lung cancer and orbital EMZL, and only one observational study has reported a case of lung cancer during a treatment‐free follow‐up of ocular adnexal EMZL.^4^

EMZL is a low‐grade lymphoma, and its prognosis is generally good, particularly for EMZL localized to the ocular adnexa.^4^ A retrospective cohort study in Japan revealed that out of 36 patients with untreated ocular adnexal EMZL, six died, of which only two deaths were due to EMZL progression.^4^ Notably, stage III lung cancer is the final opportunity for curative medical treatment. Therefore, prioritizing lung cancer treatment in our case was an appropriate strategy.

The treatment options for orbital EMZL include radiotherapy, surgery, and rituximab. In cases where radiotherapy is the preferred treatment, a common approach involves a 30 Gy/20 Fr regimen. However, if the patient has a history of extranodal marginal zone lymphoma (EMZL) and requires treatment prior to lung cancer therapy, there may be a potential delay of up to 4 weeks, which could remarkably reduce the likelihood of a successful medical cure for the lung cancer. When systemic chemotherapy is required for EMZL due to the stage, physicians typically choose treatment with rituximab plus cyclophosphamide, doxorubicin, vincristine, and prednisone; bendamustine plus rituximab; or rituximab plus cyclophosphamide, vincristine, and prednisone because the disease is classified as B‐cell lymphoma.^3^ In the present case, carboplatin plus paclitaxel was selected as the platinum‐based and taxane‐combined anticancer agent. Chemotherapy regimens, including platinum, are effective in treating patients with refractory non‐Hodgkin's lymphoma, and both lung cancer and EMZL may also respond to carboplatin. However, apart from the effectiveness of carboplatin and paclitaxel in treating lymphoma, there are two other possible reasons for the reduced tumour size seen in our case. First, steroids can temporarily reduce the size of lymphomas, and the administration of dexamethasone to prevent side effects during chemotherapy for lung cancer may have affected tumour shrinkage. However, the effect persisted after chemotherapy despite the administration of steroids only on the first day of treatment, indicating that chemotherapy was effective. Second, in some cases, ocular adnexal lymphoma can spontaneously regress,^5^ which may have occurred in the present case. However, the population studied in the previous report was distinct from our patient, and there have been no reports of the spontaneous regression of orbital EMZL. We believe that the chemotherapy for lung cancer was effective for EMZL, and therefore, treatment for lung cancer should be prioritized in cases where the two conditions occur simultaneously. The standard treatment for stage III non‐small cell lung cancer is chemoradiotherapy, followed by durvalumab. However, in this case, radiation pneumonitis was observed immediately after chemoradiotherapy, and after consultation with the patient, we decided not to administer durvalumab.

In conclusion, in cases of concurrent orbital EMZL and lung cancer, the treatment of lung cancer should take precedence.

## AUTHOR CONTRIBUTIONS

All authors contributed to the conception of the case report. Issei Oi and Minako Mori wrote the first draft. All authors revised subsequent versions and approved the final version.

## CONFLICT OF INTEREST STATEMENT

None declared.

## ETHICS STATEMENT

The authors declare that appropriate written informed consent was obtained for the publication of this manuscript and accompanying images.

## Data Availability

The data that support the findings of this study are available on request from the corresponding author. The data are not publicly available due to privacy or ethical restrictions.
